# Co-development of a clinical rehabilitation model with an evidence-based approach for torture survivors

**DOI:** 10.1192/bjo.2023.543

**Published:** 2023-08-30

**Authors:** Kolbassia Haoussou, Katy Robjant

**Affiliations:** Freedom from Torture, London, UK

**Keywords:** Torture, PTSD, trauma and stressor-related disorders, survivor empowerment

## Abstract

Torture is designed to silence, render powerless, oppress and terrify not just the individual torture survivor but the whole society where the act of torture occurs. The aftermath of torture can include trauma spectrum disorders such as post-traumatic stress disorder as well as other mental health problems, in addition to chronic pain and disability. Rehabilitation for torture survivors therefore needs to include empowerment at its heart. This is important to overturn the impact of torture on the survivor's sense of powerlessness and to address the silencing that begins with torture and is maintained by the psychological sequelae of surviving it. The organisation Freedom from Torture, together with survivors of torture, co-designed a new evidence-based clinical rehabilitation model by surveying service users and by carrying out a narrative review of the research literature and best practice guidelines. The resulting model incorporates co-delivery of therapeutic services by survivors.

The experience of torture often renders survivors unable to speak peri-traumatically as a result of para-sympathetic shut down activation and dissociation.^1^ In those who subsequently develop post-traumatic stress disorder (PTSD) or complex post-traumatic stress disorder (CPTSD), the avoidance of trauma reminders perpetuates silence around the experience of torture, resulting in a lack of societal awareness and increased likelihood of impunity for perpetrators.^2^

Effective evidence-based treatments should be offered to those who have experienced torture, to ensure the best recovery possible.

In mainstream mental health services, patient involvement in design, delivery and evaluation is long established as integral to best practice.^3^ However, meaningful patient or service user involvement is less common for refugee populations (including torture survivors). As torture survivors were deliberately silenced and disempowered, it is of critical importance to recovery that this is addressed. Freedom from Torture (FfT) (a non-governmental organisation that provides rehabilitation services) redesigned their clinical model together with survivors to ensure best-practice evidence-based rehabilitation services that meet the needs and wishes of survivors. The organisation has a strong culture of service user involvement and empowerment, following six basic principles:^4^

- recognising people as assets: transforming the perception of people from being passive recipients of care to being equal partners in designing and delivering services
- building on people's existing capabilities: actively supporting people to recognise and use their strengths
- reciprocity and mutuality: enabling those who use services to develop reciprocal relationships with professionals (and other clients), with mutual responsibilities and expectations
- peer support networks: enhancing transfer of knowledge by engaging peer networks alongside those of professionals
- breaking down barriers: minimising the distinctions between professionals and producers and consumers of services, and changing the way services are developed and provided so as to alter power dynamics
- facilitating rather than delivering: enabling professional staff and service users to become catalysts of change in delivering and receiving services.

## Method

### Design

A client survey and a scoping review were carried out. The anonymous client survey, co-designed with survivors, included questions relating to current service provision and the types of service they would like to receive. The narrative review included scrutiny of clinical trials and international guidelines regarding the appropriateness of treatments for torture survivors or similar populations according to the evidence base.

### Participants

All 483 service users receiving at least one of the services offered by FfT rehabilitation centres in the UK aged over 18 were invited to participate in the survey. The invitations were translated in the top five languages of our client group, as was the survey itself. Survivors unable to read or who spoke other languages were helped to participate via alternative arrangements. In total, 251 (52%) of the 483 clients completed the survey; 193 (77%) were men and 58 (23%) women. Clients were also able to complete the survey via smartphones. All participants provided written consent. Ethical approval was not required. However, the survey aspect of the project was reviewed by a member of the organisation's internal ethics committee.

## Results

The results showed that survivors of torture wanted to receive psychotherapy: (*n* = 223; 89%) endorsed that they would like individual therapy. Specifically, survivors wanted a treatment that focused on trauma symptoms: (*n* = 168; 67%) endorsed that they required a trauma-focused therapy, and on a different item (*n* = 193; 77%), requested a therapy that focused on PTSD symptoms.

Other areas of desired therapeutic focus were help adjusting to life in the UK (*n* = 130; 52%) and reducing social isolation (*n* = 113; 45%). Respondents also highlighted the need for physical healthcare and pain management services (*n* = 72; 29%), as well as legal and welfare support (*n* = 130; 52%) and a medico-legal report (*n* = 113; 45%).

The narrative review indicated that for the treatment of PTSD, the following therapeutic modalities were useful and recommended in various national and international guidelines (although not for torture survivors uniquely): narrative exposure therapy (NET),^5^ eye movement desensitisation and reprocessing (EMDR)^6,7^ and trauma-focused cognitive–behavioural therapy (TF-CBT).^8,9^ Historically, care of survivors was suggested to occur within a three-stage phased model.^10^

A working group of survivors and clinicians combined the results of the survey with the results of the narrative review, indicating which treatments were recommended according to the evidence base to design the new clinical model. The emerging model was further discussed with other survivors in existing forums and feedback was incorporated.

## Discussion

The co-led working group devised a rehabilitation model for torture survivors with four pillars*:* stabilisation; trauma-focused therapy; other targeted interventions; and reconnection. The ‘other targeted interventions’ pillar was included since not all survivors of torture develop PTSD and of those who do, not all prioritise treatment for it if they have comorbid conditions. Moving away from Herman's phased approach,^10^ we determined that all four pillars of treatment could be offered from the outset (sometimes in parallel), and that this should depend on the wishes and needs of the individual survivor. All survivors who met diagnostic criteria at assessment would be offered an evidence-based treatment for PTSD as first choice.

### Stabilisation through co-delivery with survivors

Stabilisation phases of therapy typically involve supporting survivors with the practical and psychological scaffolding required to achieve basic safety in all regards. Psychoeducation about the nature of trauma and effective treatments available is provided to increase motivation for engagement. Ensuring that empowerment was maximised, we proposed the inclusion of survivors in the delivery of stabilisation; this involved their informing other survivors about services available at a ‘welcome day’, a proposed buddying system of support that remains throughout the client journey, and the co-delivery of psychoeducation and symptom management groups. At the outset of their therapeutic journey, new survivors meet other survivors who have recovered, fostering hope and trust.

### Trauma-focused therapies

Our clinical model includes three evidence-based approaches. With currently the strongest evidence base for survivors of multiple traumas, NET is attractive as it has been trialled in over 30 countries with different populations and is widely culturally applicable.^11^ The treatment includes a human rights focus and documentation of atrocities endured through the production of therapeutic ‘testimony’. This approach can also be delivered by non-expert health professionals. As alternative evidence-based approaches, EMDR and TF-CBT are offered, according to survivor choice.

### Other targeted interventions

Guided by survivor choice, additional evidence-based therapies were included in the model to be offered for non-PTSD-related psychological difficulties, either prior or subsequent to trauma-focused therapy. A range of approaches are offered according to diagnosis and appropriate recommendations for those conditions.

### Reintegration and reconnection

Survivors will co-lead resilience-based workshops (following narrative methodologies, including the ‘tree of life’)^12^ prior to discharge from therapy and prior to engaging in longer-term peer support. Survivor activism, which is not motivated by rehabilitation goals, may still have rehabilitative benefits and may help to overcome silencing and powerlessness instilled by torture.

### Implications

The rehabilitation needs of torture survivors include psychological therapies across four ‘pillars', treatment of physical pain and help accessing the healthcare system, as well as legal protection and welfare support. Our introduction of co-delivered rehabilitation services in the stabilisation and reconnection phases of treatment suggests that there is the potential to trial survivor-led delivery of trauma-focused therapy such as NET. Survivor involvement at the highest level in design and delivery of rehabilitation services is key to empowerment following torture.

## Data Availability

Survey results are available on request from the corresponding author.
